# Supplementary material for: Does Constitutive Expression of Defense-Related Genes and Salicylic Acid Concentrations Correlate with Field Resistance of Potato to Black Scurf Disease?
Source: Bioengineering (Basel). 2023 Oct 24;10(11):1244. doi: 10.3390/bioengineering10111244 (PMC10669363; doi:10.3390/bioengineering10111244)
Supplement: Supplementary file 1 [file bioengineering-10-01244-s001.zip › bioengineering-2626023-supplementary.pdf]

Table S1. Oligonucleotide primer sets.

Gene	Function	Forward primer	Reverse primer	Accession	Reference
PR1	Pathogenesis-related 1	GGTGCAGGAGAGAACCTT	GGTACCATAGTTGTAGTTTGGCT	AJ250136	[1]
PR2	1,3-β-Glucan glucanohydrolase	CACATTGCTTCTGGGATGGA	AACATCTGGCCAGAAATCTTTAA	AF067863	[2]
PR3	Acidic endochitinase	ATGGCTGCCTTTTTCGGTCA	TACCTTGTCCAGCTCGTTCG	NM_001318545	[1]
PR6	Proteinase inhibitor II	TGCCCACGTTCAGAAGGAAG	TGGGTCAGATTCTCCTTCGC	KX710107	[1]
PR10	Pathogenesis-related STH-2-like	TGATGTTAAGAGCATTGAGGTTGT	ATTGGACCACCTTCAACAAAGTT	XM_006340827	[1]
PAL	Phenylalanine ammonia-lyase	TCGAGGACGAATTGAAGGCAA	GCACATTGCTGTGAACACCTT	MH636300	[1]
ICS	Isochorismate synthase	CTTCTCCGGTCGAAGAGTTG	TGAAAAGGGGCGTAAATGAG	XM_015312034	this study
ACT	Reference gene actin	GCTTCCCGATGGTCAAGTCA	GGATTCCAGCTGCTTCCATTC	X55749	[1]

References

1. Genzel, F.; Franken, P.; Witzel, K.; Grosch, R. Systemic induction of salicylic acid-related plant defences in potato in response to *Rhizoctonia solani* AG3PT. *Plant Pathology* **2018**, *67*, 337-348, doi:https://doi.org/10.1111/ppa.12746.
2. Lehtonen, M.J.; Somervuo, P.; Valkonen, J.P.T. Infection with *Rhizoctonia solani* induces defense genes and systemic resistance in potato sprouts grown without light. *Phytopathology* **2008**, *98*, 1190-1198, doi:https://doi.org/10.1094/phyto-98-11-1190.
